# Lycopene Protects Deoxynivalenol-Induced Intestinal Barrier Dysfunction and NLRP3 Inflammasome Activation by Targeting the ERK Pathway

**DOI:** 10.3390/antiox14121513

**Published:** 2025-12-17

**Authors:** Zihui Cai, Zhi Lu, Youshuang Wang, Wenxi Song, Xu Yang, Cong Zhang

**Affiliations:** 1 College of Veterinary Medicine, Henan Agricultural University, Zhengzhou 450002, China; zihuicai@stu.henau.edu.cn (Z.C.); yswang@stu.henau.edu.cn (Y.W.); songwx@stu.henau.edu.cn (W.S.); 2 Department of Psychological and Cognitive Sciences, Tsinghua University, Beijing 100084, China; luzhi@tsinghua.edu.cn; 3 Institute for Brain and Cognitive Sciences, Tsinghua University, Beijing 100084, China

**Keywords:** IPEC-J2 cells, deoxynivalenol, lycopene, MAPK pathway, NLRP3 inflammasome

## Abstract

In agricultural production, Deoxynivalenol (DON) generally exists and contaminates wheat, corn, and other grains, causing intestinal damage and immunotoxicity. Lycopene (LYC), an antioxidant, anti-inflammatory carotenoid, is mainly found in red fruits such as tomatoes and has been investigated for its great medicinal advantages. This study aimed to investigate the protective effect of LYC against DON-induced enterotoxicity. Our findings demonstrated that incubation of IPEC-J2 cells with 0.5 μM DON for 24 h caused intestinal barrier impairment and oxidative stress induction, which subsequently led to increased secretion of pro-inflammatory factors (TNF-α, IL-1β, IL-18, and IL-6) and decreased secretion of the counterregulatory factor (IL-10). Furthermore, DON ultimately induced NLRP3 inflammasome activation through the stimulation of the MAPK/NF-κB pathway. It is worth mentioning that the above changes were reversed after adding 30 μg/mL of LYC to DON-exposed IPEC-J2 cells. In addition, further experiments confirmed that ERK activator (4-Methylbenzylidene camphor, 4-MBC) eliminated the positive effect of LYC on alleviating enterotoxicity induced by DON in IPEC-J2 cells. In addition, further experiments confirmed that 4-MBC eliminated the positive effect of LYC on alleviating enterotoxicity induced by DON. In general, our study certified that ERK is a therapeutic target for LYC protecting DON-induced intestinal barrier dysfunction and NLRP3 inflammasome activation.

## 1. Introduction

Mycotoxins are secondary metabolites that can develop at any stage of grain production [1]. Deoxynivalenol (DON) is the most common mycotoxin produced by *Fusarium* in agriculture [2]. It is reported that the detection rate of DON increased from 46% (2005) to 64% (2008) in a total of 17,316 samples, mostly collected from Europe and Asia [3]. Another decade-long (from 2008 to 2017) research demonstrated that DON was mostly detected in wheat and barley (65% and 61%, respectively), covering 74,821 samples from 100 countries [4]. What’s worse, a comprehensive quantitative modeling method predicted that the advance of wheat flowering time in the next few decades would increase the concentration and pollution range of DON [5]. In addition, DON exposure is strongly related to human and animal health, and different species (pigs/human  >  mice/rats  >  poultry) have varying sensitivities due to differences in toxicokinetics and metabolism [6]. DON-exposed food will induce various toxic effects characterized mainly by intestinal damage and ultimately harm animal health [7].

The gut is the first barrier to protect animals from food contaminants [8]. DON exposure can cause diarrhea, vomiting, immune disorders, and intestinal inflammation [9]. There have been many studies on DON-induced enterotoxicity in recent years. Oral ingestion is the main way for DON to enter the gastrointestinal tract, leading to damage to intestinal epithelial cell absorption function and further causing enterotoxicity [10]. Many studies indicated that DON can induce enterotoxicity by causing intestinal damage (e.g., oxidative damage, mitochondrial dysfunction, apoptosis, and gut microbiota imbalance), with relevant evidence from both in vitro and in vivo experiments—such as 0.5 μM DON treating cells for 24 h [11], and 3.8 mg/kg DON supplemented in pig feed with ad libitum feeding for 28 days [12]. Interestingly, DON could also damage intestinal cells and the barrier via activating the necroptosis pathway [13]. A study revealed that even low doses of DON intake can exacerbate intestinal inflammation and damage the epithelial barrier in piglets [14]. Another study indicated that non-toxic doses of DON can aggravate the development of colitis through the JAK2/STAT3 pathway [15]. The latest research confirmed that DON induced intestinal barrier damage in both intestinal porcine jejunum epithelial cells (IPEC-J2 cells) and mouse jejunum tissue [16]. DON can depress mucins and antimicrobial peptides secretion, activate cytokine production, and alter gut microflora composition to further aggravate enterotoxicity [17]. Furthermore, in IPEC-J2 cells, the inflammatory response triggered by deoxynivalenol (DON) through activating the TNF-α/NF-κB/MLCK signaling pathway is an important factor leading to the impairment of the intestinal barrier [18]. To date, many mechanisms of DON-induced enterotoxicity have been discovered. Despite this, the molecular processes behind DON-induced intestinal damage are largely undetermined. Our previous study demonstrated that LYC could mediate mitochondrial homeostasis to alleviate DON-induced intestinal barrier damage. Building on this, the present in vitro study used IPEC-J2 cells to further explore LYC’s barrier-protective mechanisms, with DON exposure at 0.5 μM for 24 h and LYC treatment at 30 μg/mL. We employed cell permeability assays and immunofluorescence staining to evaluate intestinal barrier function and combined with molecular biology techniques to dissect the underlying signaling pathways.

The regulation of gut immune response and epithelial barrier permeability is important for the maintenance of intestinal homeostasis. Once the homeostasis is broken, it can result in inflammation and harm gut health [19,20]. NLRP3 inflammasome, as a significant sensor in the immune system, can capture danger signals and recruit Caspase-1. After that, the release of interleukin (IL)-1β and IL-18 can be promoted by activated Caspase-1 [21]. A review summarized that DON mediated NLRP3 inflammasome activation in various cells, and DON can cause intestinal mechanical/immune barrier dysfunction and increase the expression of inflammasome-related factors under the infection of enterotoxigenic *Escherichia coli* (ETEC) and Porcine epidemic diarrhea virus (PEDV) [22]. Our previous research reported that NLRP3 inflammasome activation is a key target of DON-induced IPEC-J2 cells inflammation [23]. Moreover, the pathways of mitogen-activated protein kinase (MAPK) and nuclear factor-kappa B (NF-κB) can also regulate inflammation [24]. MAPK signals are triggered by extracellular signal-regulated kinase (ERK), JUN N-terminal kinase (JNK), and P38 kinase [25]. Studies have confirmed that DON can upgrade the release of inflammatory factors by MAPK pathway activation [26]. Interestingly, the increase in inflammatory cytokines via the MAPK pathway will cause cytotoxicity, which is related to NF-κB transcription [27]. Furthermore, DON can aggravate LPS-induced intestinal barrier injury and inflammation through NF-κB/LC3 signal pathway activation [28]. Therefore, targeting NLRP3 inflammasome activation and inflammation suppression may be an attractive strategy for treating DON-induced enterotoxicity or related intestinal diseases.

Lycopene (LYC), as a natural plant extract, is mainly found in tomatoes as well as other red fruits or vegetables. LYC has been found to have strong antioxidant and anti-inflammatory properties and has been studied for more than 70 years [18,29,30]. Recently, a large number of research studies at home and abroad have shown the strong protective effect of LYC in the gut. Specifically, LYC pre-treatment (at 50 mg/kg supplemented in pig diets for 28 days) has been shown to protect the intestinal barrier and improve gut microbiota by regulating the Nrf2-Keap1 pathway [31]. Additionally, LYC can regulate the NF-κB pathway, thereby decreasing the incidence of inflammatory bowel disease (IBD) [32]. In addition, a similar study has shown that LYC can alleviate colitis by improving intestinal function and inhibiting *Escherichia coli* adhesion [33]. Further research found that in 101,680 U.S. adults, LYC may be associated with a 20 percent lower risk of colorectal cancer [34]. It is reported that LYC can alleviate Fumonisin B1 (FB1)-induced mitochondrial structure damage and loss of mitochondrial function in chicken hepatocytes [35]. Our previous research results have shown that LYC can significantly reduce the accumulation of ROS and malondialdehyde (MDA) levels caused by DON, while also maintaining mitochondrial function, thereby alleviating intestinal damage caused by oxidative stress [36]. However, whether LYC can relieve DON-induced enterotoxicity through regulating NLRP3 inflammasome and MAPK pathway is unknown. Notably, to specifically verify the role of the ERK subfamily (a key branch of the MAPK pathway) in LYC’s protective mechanism, 4-Methylbenzylidene camphor (4-MBC) is a convenient tool: it acts as a specific activator of the ERK pathway, which exerts its effect by promoting the phosphorylation of ERK1/2 without affecting other MAPK subfamilies (e.g., JNK, p38) [37]. This specificity supports that changes in DON-induced enterotoxicity following 4-MBC intervention are likely associated with ERK pathway modulation, which helps reduce potential interference from non-target MAPK branches (e.g., JNK, p38). Hence, the IPEC-J2 cells model in this article was used to investigate the therapeutic function of LYC on DON-induced enterotoxicity and deeply explore the regulatory mechanism of the MAPK pathway in this process.

## 2. Materials and Methods

### 2.1. Cell Culture and Treatment

IPEC-J2 cells were originally bought from Tongpai Biotechnology Co., Ltd. (Shanghai, China) and incubated with DMEM (G4511, Servicebio, Wuhan, China) medium in a humidified incubator (37 °C with 5% CO_2_). The following components: 15% FBS (Sijiqing, Hangzhou, China), 1% penicillin-streptomycin (Servicebio, Wuhan, China), and 1% glutamine (Servicebio, Wuhan, China) were added to the DMEM medium.

DON was purchased from Pribolab Biotech Co., Ltd. (MSS1011, Qingdao, China), and LYC was purchased from Solarbio Science and Technology Co., Ltd. (IL0510, Beijing, China). Based on previous studies, the optimal non-cytotoxic concentrations of DON (0.5 μM) and LYC (30 µg/mL) have been determined [12,36]. When the cells confluence reached 70–80%, cells were treated with DON (0.5 μM) and LYC (30 µg/mL) for 24 h at 37 °C and 5% CO_2_. Next, SB203580 (HY-10256, MCE, Monmouth Junction, NJ, USA), SCH772984 (HY-50846, MCE, Monmouth Junction, NJ, USA), and SP600125 (HY-12041, MCE, Monmouth Junction, NJ, USA), which are p38, ERK, and JNK inhibitors, respectively, were used to ascertain the intestinal injury-inhibiting pathway of DON in IPEC-J2 cells. In addition, 4-MBC, which is an ERK activator, was purchased from MCE (HY-17587, MCE, Monmouth Junction, NJ, USA).

### 2.2. Cell Viability Detection

2 × 10^4^ cells were counted by cell counting plate and inoculated into 96-well plates, with 5 repeat groups set up. After treatment with different drugs, CCK-8 reagent (K1018, APExBIO, Houston, TX, USA) was added to every experimental well. After 2 h of inoculation, the optical densities (O.D.) were detected by the Infinite M200 FA plate reader (Tecan Group Ltd., Männedorf, Switzerland) at 450 nm.

### 2.3. Immunofluorescence

A total of 2 × 10^4^ cells were counted using a cell-counting plate and inoculated into 24-well plates. When the cells confluence reached 70–80%, cells were treated with different drugs for 24 h. Subsequently, the cells were rinsed with PBS and then fixed with 4% paraformaldehyde for 15 min. After that, the Quick Block™ Blocking Buffer was blocked for 15 min. Next, the cells were incubated with primary antibodies Claudin-1 (1:200, Wanleibio, Shenyang, China) and Occludin (1:150, Wanleibio, Shenyang, China) at 4 °C. After incubating overnight, the cells were incubated with second antibodies conjugated to FITC (K1203, APExBIO, Houston, TX, USA) and Cy3 (K1209, APExBIO, Houston, TX, USA) for 1 h, respectively. After staining with the 4′,6-diamidino-2-phenylindole (DAPI) dye for 5 min, fluorescence intensity was detected using the EVOS M5000 cell imaging system (ThermoFisher, Waltham, MA, USA).

### 2.4. Cell Permeability Assay

IPEC-J2 cells permeability was assessed using Transwell-24 well permeable support system (14311-D, Labselect, Beijing, China). After preincubating the inserts with culture medium for 1 h, 2 × 10^4^ cells were seeded on the insert membranes and incubated with culture medium for about 24 h at 37 °C. Then different drugs were added, and after 24 h of inoculation, all transwell inserts were gently rinsed with PBS. Next, the upper chamber was filled with 200 μL fluorescein (Uranine) sodium (MCE, HY-D0208, Monmouth Junction, NJ, USA), while the lower chamber was filled with 600 μL PBS. After incubating for 2 h, PBS in the lower chamber was transferred to a 96-well plate, and the intensity of the dye was measured at 490 nm with the Infinite M200 FA plate reader.

### 2.5. Enzyme-Linked Immunosorbent Assay (ELISA)

A total of 2 × 10^5^ cells were counted using a cell-counting plate and inoculated into 6-well plates. When cell confluence reached 70–80%, cells were treated with different drugs. After 24 h of treatment, the medium supernatants were all collected, respectively. Then, the inflammatory cytokine levels (TNF-α, IL-6, IL-10, IL-18, and IL-1β) were detected using ELISA kits. Porcine-specific ELISA kits for IL-1β (JLC9772), IL-6 (JLC9784), IL-18 (JLC9769), and TNF-α (JLC9892) were purchased from Shanghai Jingkang Bioengineering Co., Ltd. (Shanghai, China). Finally, supernatants were treated according to the assay instructions, and the O.D. was detected at 450 nm by the Infinite M200 FA plate reader.

### 2.6. Detection of Oxidative Stress Markers

The contents of ROS (E004-1-1), H_2_O_2_ (A064-2-1), and GSH (A006-2-1), total antioxidant capacity (A015-2-1), catalase (CAT) activity (A007-1-1), and superoxide dismutase (SOD) activity (A001-3) in cells were measured by a commercial kit (Nanjing Jiancheng Bioengineering Institute, Nanjing, China) according to the manufacturer’s instructions.

### 2.7. Quantitative Real-Time PCR (qRT-PCR)

A total of 2 × 10^5^ cells were counted by cell counting plate and inoculated into 6-well plates. When cell confluence reached 70–80%, cells were treated with different drugs. After 24 h of treatment, the cells’ RNA was extracted with the RNA extraction solution. qRT-PCR was performed on the qTOWER3G real-time PCR detection system using ChamQ Universal SYBR qPCR Master Mix. As shown in Table S1, the primer sequences were synthesized by Tsingke Biotechnology Co., Ltd. (Beijing, China). The relative mRNA expressions were calculated by the 2^−ΔΔCt^ method compared with GAPDH.

### 2.8. Western Blot Analysis

After normal passage of the cells in 75 cm^2^ cell culture dishes until the cell confluence reached 70–80%; different drugs were added. After 24 h of treatment, proteins were extracted using the Nuclear and Cytoplasmic Protein Extraction Kit (Beyotime, Shanghai, China). The protein was transferred to a PVDF membrane after being separated via SDS gel electrophoresis. Then, ERK 1/2 (A4782, ABclonal, Wuhan, China), P-ERK 1/2 (AP0485, ABclonal, Wuhan, China), JNK 1/2/3 (A4867, ABclonal, Wuhan, China), P-JNK (AP0631, ABclonal, Wuhan, China), p38 (A4771, ABclonal, Wuhan, China), P-p38 (AP0056, ABclonal, China), NLRP3 (WL02635, Wanleibio, Shenyang, China), Caspase-1 (WL02996, Wanleibio, Shenyang, China), ASC (WL02462, Wanleibio, Shenyang, China), IL-1β (A19635, ABclonal, Wuhan, China), IL-18 (WL01127, Wanleibio, Shenyang, China), NF-κB-p65 (A19653, ABclonal, Wuhan, China), P-NF-κB-p65 (WL02169, Wanleibio, Shenyang, China), Lamin B (WL01775, Wanleibio, Shenyang, China), β-actin (GB11001, Servicebio, Wuhan, China), and secondary antibody (GB23303, Servicebio, Wuhan, China) were incubated. Each band was monitored using the Amersham Imager 600 (Tanon Science & Technology Co., Ltd., Shanghai, China).

### 2.9. Data Statistics and Analysis

Statistical analysis was performed using GraphPad Prism software (version 9.5.0, GraphPad Software, San Diego, CA, USA). All experimental data were presented by mean ± SEM. Analysis of all data was conducted by one-way ANOVA, *p* < 0.05, indicating that the results are statistically significant. (*) showed the significance of differences between the CON group and another group (* *p* < 0.01, ** *p* < 0.01). (#) showed the significance of differences between DON and LYC + DON (# *p* < 0.01, ## *p* < 0.01). ($) showed the significance of differences between LYC + DON and LYC + DON + 4-MBC ($ *p* < 0.01, $$ *p* < 0.01).

## 3. Results

### 3.1. LYC Alleviated DON-Induced Intestinal Epithelial Barrier Impairment and Oxidative Stress in IPEC-J2 Cells

**Figure 1:**
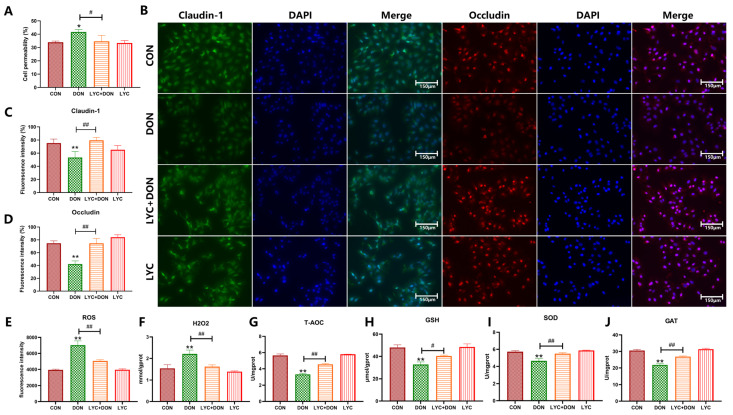
LYC alleviated DON-induced intestinal epithelial barrier impairment in IPEC-J2 cells. (**A**) Analysis of cell permeability. (**B**) Changes in immunofluorescence. Scale bar = 150 μm (applies to all separate red/green channel images and merged images in this figure, captured under ×200 magnification). (**C**) Analysis based on Claudin-1 green fluorescence intensity in (**B**). (**D**) Analysis based on Occludin red fluorescence intensity in (**B**). (**E**) ROS content. (**F**) H_2_O_2_ content. (**G**) T-AOC level. (**H**) GSH content. (**I**) SOD activity. (**J**) CAT activity. IPEC-J2 cells were divided into CON, DON, LYC + DON, and LYC groups. Each reported value represents the mean ± SEM. (*) for the significance of differences between the control and another, * *p* < 0.05, ** *p* < 0.01; (#) for the significance of differences between DON and LYC + DON, # *p* < 0.05, ## *p* < 0.01.

Our previous study demonstrated that LYC could mediate mitochondrial homeostasis to alleviate DON-induced intestinal barrier damage. In this study, we used a cell permeability assay and immunofluorescence staining to further explore the barrier protection of LYC at the cellular level. The results of the experiment have shown that compared with the CON group, DON significantly increased cell permeability, and the addition of LYC reversed this change (*p* < 0.05) (Figure 1A). Immunofluorescence staining of Claudin-1 and Occludin revealed that DON significantly diminished the fluorescence intensity than in CON group. Furthermore, LYC addition enhanced the fluorescence intensity compared to the DON group (*p* < 0.01) (Figure 1B–D). These data demonstrated that DON could result in an increase in cell permeability and damage the intestinal barrier, while LYC can alleviate intestinal barrier injury in DON-induced IPEC-J2 cells. Compared with the CON group, DON significantly increased the content of ROS and H_2_O_2_ (*p* < 0.01), LYC treatment reversed the increased content of ROS and H_2_O_2_ (*p* < 0.01) (Figure 1E,F); Compared with the CON group, DON significantly decreased the T-AOC and GSH, and the activities of SOD and CAT (*p* < 0.01) (Figure 1G–J), while LYC treatment reversed the change in DON on T-AOC, GSH, SOD, and CAT (*p* < 0.01). These results indicated that LYC can reverse the IPEC-J2 cells’ oxidative stress induced by DON.

### 3.2. LYC Alleviated DON-Induced Inflammatory Injury and NF-κB Translocation in IPEC-J2 Cells

**Figure 2:**
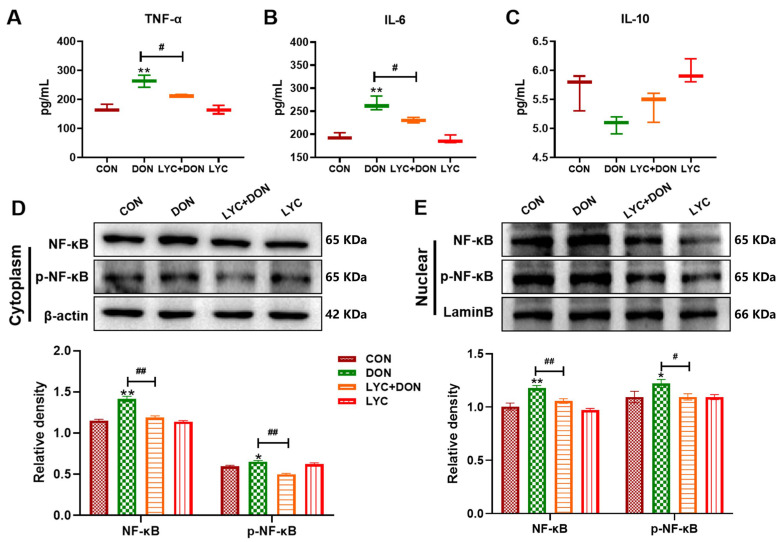
LYC alleviated DON-induced inflammatory injury and NF-κB translocation in IPEC-J2 cells. (**A**) ELISA analysis of TNF-α. (**B**) ELISA analysis of IL-6. (**C**) ELISA analysis of IL-10. (**D**) Western blot analysis of NF-κB and p-NF-κB proteins in the cytoplasm. (**E**) Western blot analysis of NF-κB and p-NF-κB proteins in the nucleus. IPEC-J2 cells were divided into CON, DON, LYC + DON, and LYC groups. Each reported value represents the mean ± SEM. (*) for the significance of differences between the control and another, * *p* < 0.05, ** *p* < 0.01; (#) for the significance of differences between DON and LYC + DON, # *p* < 0.05, ## *p* < 0.01.

In the IPEC-J2 cells model, the effects of LYC on NF-κB translocation and inflammatory damage generated by DON were examined using Western Blot and an ELISA kit. The results are displayed in Figure 2. In the DON group, the contents of inflammatory cytokines (TNF-α and IL-6) were higher (*p* < 0.01), and the counterregulatory factor (IL-10) was lower than in the CON group (Figure 2A–C). Furthermore, DON enhanced the nuclear and cytoplasmic protein expressions of NF-κB-p65 and P-NF-κB-p65 (*p* < 0.05, *p* < 0.01) (Figure 2D,E). On the other hand, DON-induced NF-κB-p65 nuclear translocation and associated inflammatory factor production were decreased (*p* < 0.05, *p* < 0.01) by LYC therapy. These findings showed that in IPEC-J2 cells, LYC can reduce inflammatory damage and prevent DON-induced NF-κB-p65 nuclear translocation.

### 3.3. LYC Inhibited DON-Induced the Activation of NLRP3 Inflammasome in IPEC-J2 Cells

**Figure 3:**
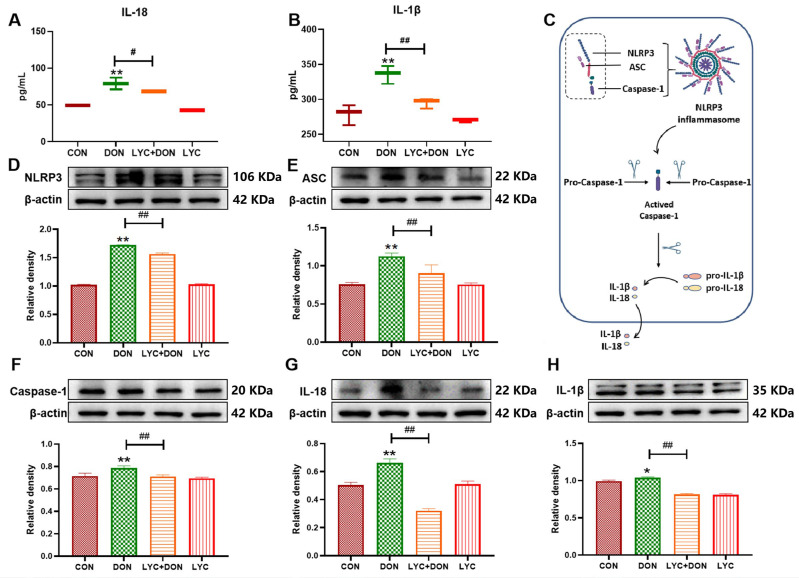
LYC inhibited DON-induced activation of the NLRP3 inflammasome in IPEC-J2 cells. (**A**) ELISA analysis of IL-18. (**B**) ELISA analysis of IL-1β. (**C**) The mechanism diagram of the NLRP3 inflammasome. (**D**) Western blot analysis of NLRP3 protein. (**E**) Western blot analysis of ASC protein. (**F**) Western blot analysis of Caspase-1 protein. (**G**) Western blot analysis of IL-18 protein. (**H**) Western blot analysis of IL-1β protein. IPEC-J2 cells were divided into CON, DON, LYC + DON, and LYC groups. Each reported value represents the mean ± SEM. (*) for the significance of differences between the control and another, * *p* < 0.05, ** *p* < 0.01; (#) for the significance of differences between DON and LYC + DON, # *p* < 0.05, ## *p* < 0.01.

NLRP3 inflammasome and downstream inflammatory cytokine secretion were determined by Western blot, qRT-PCR, and an ELISA kit. Compared with the CON group, the secretion of IL-18 and IL-1β was significantly improved in the DON group (*p* < 0.01) (Figure 3A,B). The mechanism diagram of the NLRP3 inflammasome is shown in Figure 3C. In addition, NLRP3 and its downstream inflammatory factors Caspase-1, ASC, IL-1β, and IL-18 (*p* < 0.05, *p* < 0.01) (Figure 3D–H) protein expressions were all up-regulated under DON exposure. These results were all reversed after LYC treatment (*p* < 0.01), suggesting that LYC can inhibit NLRP3 inflammasome activation in DON-induced IPEC-J2 cells.

### 3.4. LYC Inhibited DON-Induced the Activation of the MAPK Signaling Pathway in IPEC-J2 Cells

**Figure 4:**
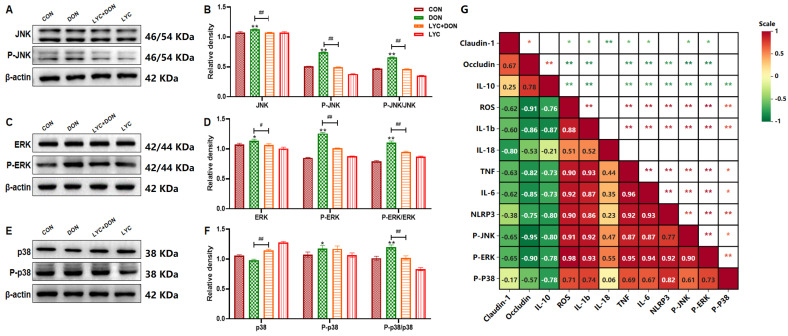
LYC inhibited DON-induced activation of the MAPK signaling pathway in IPEC-J2 cells. (**A**) Western blot bands of JNK and P-JNK. (**B**) Western blot analysis of JNK and P-JNK proteins, (**C**) Western blot bands of ERK and P-ERK. (**D**) Western blot analysis of ERK and P-ERK proteins. (**E**) Western blot bands of p38 and P-p38. (**F**) Western blot analysis of p38 and P-p38 proteins. IPEC-J2 cells were divided into CON, DON, LYC + DON, and LYC groups. (**G**) Correlation heatmap analysis: Color (red→green) indicates positive→negative correlation intensity among intestinal barrier proteins, inflammatory factors, and MAPK pathway proteins. Each reported value represents the mean ± SEM. (*) for the significance of differences between the control and another, * *p* < 0.05, ** *p* < 0.01; (#) for the significance of differences between DON and LYC + DON, # *p* < 0.05, ## *p* < 0.01.

To determine whether the MAPK pathway was involved in LYC mitigating DON-induced IPEC-J2 cells injury, we used Western Blot to detect MAPK pathway-related factors. Our data indicated that both P-ERK/ERK and P-JNK/JNK protein expressions were significantly increased in the DON group than those of the CON group (*p* < 0.05, *p* < 0.01) (Figure 4A–D). However, the expression of p38 in the DON group was lower than the CON group, while P-p38 and their ratio were significantly higher (*p* < 0.05, *p* < 0.01) (Figure 4E,F). In addition, LYC treatment could significantly inhibit the activation of the MAPK pathway induced by DON (*p* < 0.05, *p* < 0.01). The correlation analysis (Figure 4G) further demonstrated the associations between the indicators related to intestinal barrier function, oxidative stress, inflammatory response, and MAPK pathway activation. In summary, the results suggested that LYC can inhibit the activation of the MAPK signal pathway caused by DON in IPEC-J2 cells.

### 3.5. Protective Effect of LYC on Intestinal Barrier Is Mediated by ERK in the MAPK Signaling Pathway

**Figure 5:**
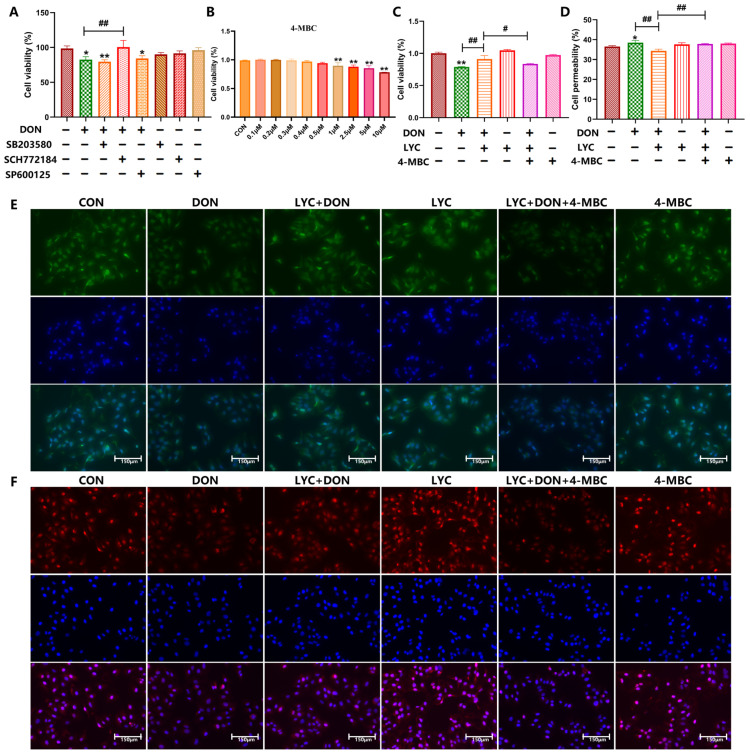
The protective effect of LYC on the intestinal barrier is mediated by ERK in the MAPK signaling pathway. (**A**) Changes in cell viability under DON and MAPK inhibitors. (**B**) Changes in cell viability under different concentrations of 4-MBC (0.1, 0.2, 0.3, 0.4, 0.5, 1, 2.5, 5, and 10 μM). (**C**) Changes in cell viability of LYC (30 μg/mL), DON (0.5 μM), and 4-MBC (0.3 μM). (**D**) Changes in cell permeability. (**E**) Green fluorescence intensity of Claudin-1. (**F**) Red fluorescence intensity of Occludin. Scale bar = 150 μm (applies to all separate red/green channel images and merged images in this figure, captured under ×200 magnification). IPEC-J2 cells were divided into CON, DON, LYC + DON, LYC, LYC + DON + 4-MBC, and 4-MBC groups. Each reported value represents the mean ± SEM. (*) for the significance of differences between the control and another, * *p* < 0.05, ** *p* < 0.01; (#) for the significance of differences between the DON and LYC + DON, # *p* < 0.05, ## *p* < 0.01.

The above data confirmed that LYC can protect the intestinal barrier and reduce inflammatory injury by regulating the MAPK signaling pathway caused by DON in IPEC-J2 cells. Next, we determined the effects of MAPK inhibitors, including p38 (SB203580), ERK (SCH772184), and JNK (SP600125) inhibitors, on DON-induced injury of the intestinal barrier and inflammation. The result was that the ERK inhibitor SCH772184 was able to reverse the DON-induced decline in cell viability compared to the DON group (*p* < 0.01) (Figure 5A). This suggested that DON exerts cytotoxic effects through the ERK signaling pathway. Therefore, we used ERK activators (4-MBC) to reverse verify whether LYC could alleviate DON-induced cell damage through the ERK signaling pathway.

We first selected the optimal concentration of 4-MBC. Briefly, cells were treated with different concentrations of 4-MBC (0, 0.1, 0.2, 0.3, 0.4, 0.5, 1, 2.5, 5, and 10 μM). The results of cell viability showed that compared with the CON group, 0.1–0.3 μM 4-MBC had no effect on cells while the cell viability was decreased from 0.4 and significantly decreased from 1 to 10 μM (*p* < 0.01) (Figure 5B). Thus, we chose 0.3 μM 4-MBC for the subsequent experiment and divided the experiment into six groups: the CON group (vehicle-free CON medium), the DON group (0.5 μM DON), the LYC + DON group (30 µg/mL LYC + 0.5 μM DON), the LYC group (30 µg/mL LYC), the LYC + DON + 4-MBC group (30 µg/mL LYC + 0.5 μM DON + 0.3 μM 4-MBC), and the 4-MBC group (0.3 μM 4-MBC). The results showed that DON decreased cell viability (*p* < 0.01) (Figure 5C), fluorescence intensity of Claudin-1 (Figure 5E) and Occludin (Figure 5F), while increasing cell permeability (*p* < 0.05) (Figure 5D). The above changes were reversed by LYC (*p* < 0.01). In addition, the effect of LYC was reduced after 4-MBC intervention (*p* < 0.05, *p* < 0.01). To sum up, 4-MBC reversed the therapeutic effect of LYC on DON-induced IPEC-J2 cells damage, which also suggested that LYC can protect IPEC-J2 cells through the ERK signal pathway.

### 3.6. LYC Alleviated DON-Induced Inflammatory Injury and NF-κB Translocation by Targeting ERK in IPEC-J2 Cells

**Figure 6:**
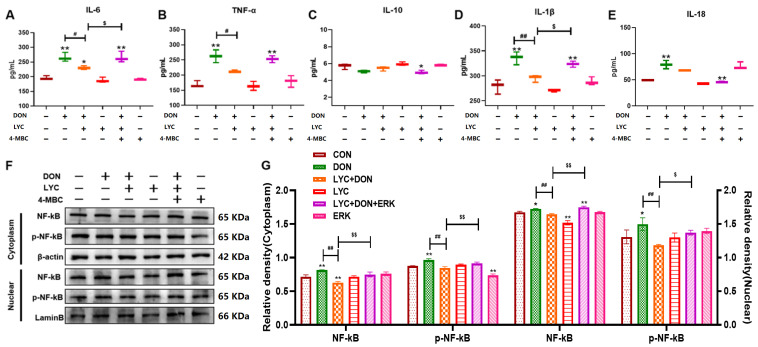
LYC alleviated DON-induced inflammatory injury and NF-κB translocation by targeting ERK in IPEC-J2 cells. (**A**) ELISA analysis of IL-6. (**B**) ELISA analysis of TNF-α. (**C**) ELISA analysis of IL-10. (**D**) ELISA analysis of IL-1β. (**E**) ELISA analysis of IL-18. (**F**) Western blot bands of NF-κB and p-NF-κB in the cytoplasm and nucleus. (**G**) Western blot analysis of NF-κB and p-NF-κB proteins in the cytoplasm and nucleus. IPEC-J2 cells were divided into CON, DON, LYC + DON, LYC, LYC + DON + 4-MBC, and 4-MBC groups. Each reported value represents the mean ± SEM. (*) for the significance of differences between the control and another, * *p* < 0.05, ** *p* < 0.01; (#) for the significance of differences between DON and LYC + DON, # *p* < 0.05, ## *p* < 0.01. ($) for the significance of differences between LYC + DON and LYC + DON + 4-MBC, $ *p* < 0.05, $$ *p* < 0.01.

The changes in inflammatory cytokines, as well as NF-κB in both cytoplasm and nucleus, were all examined in IPEC-J2 cells. As shown in Figure 6, DON increased the release of pro-inflammatory cytokines IL-6, TNF-α, IL-1β, and IL-18 (*p* < 0.01) (Figure 6A,B,D,E) and decreased the release of the counterregulatory factor IL-10 (Figure 6C). These changes were reversed after LYC addition (*p* < 0.05, *p* < 0.01). However, the effect of LYC was reduced after 4-MBC intervention (*p* < 0.05). In addition, LYC mitigated the DON-induced protein expressions of NF-κB and *p*-NF-κB in both nucleus and cytoplasm, while 4-MBC intervention reduced the mitigative effect of LYC (*p* < 0.05, *p* < 0.01) (Figure 6F,G). These results suggested that LYC treatment could attenuate NF-κB translocation and inflammatory injury by targeting ERK in DON-induced IPEC-J2 cells.

### 3.7. LYC Inhibited DON-Induced NLRP3 Inflammasome Activation by Targeting ERK in IPEC-J2 Cells

**Figure 7:**
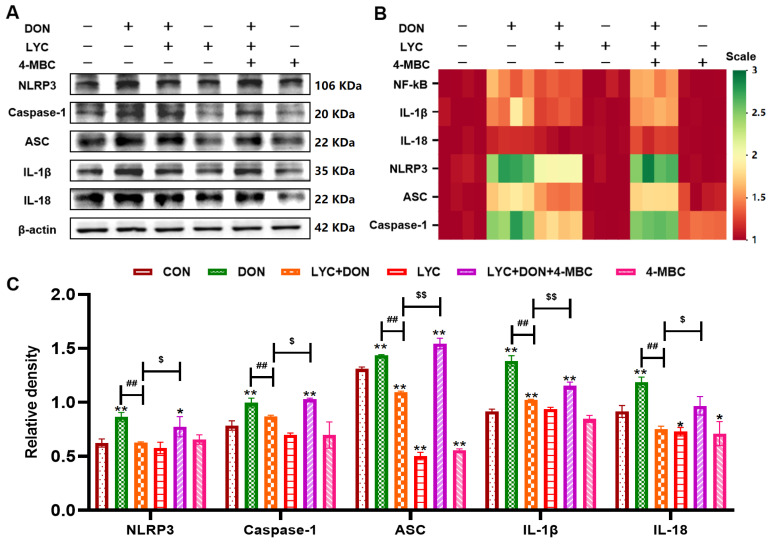
LYC inhibited DON-induced NLRP3 inflammasome activation by targeting ERK in IPEC-J2 cells. (**A**) Western-blot bands of NLRP3 inflammasome—related proteins. (**B**) Relative RNA abundance of NLRP3 inflammasome—associated factors. (**C**) Western blot analysis of NLRP3 inflammasome—related proteins. IPEC-J2 cells were divided into CON, DON, LYC + DON, LYC, LYC + DON + 4-MBC, and 4-MBC groups. Each reported value represents the mean ± SEM. (*) for the significance of differences between the control and another, * *p* < 0.05, ** *p* < 0.01; (#) for the significance of differences between the DON and LYC + DON, ## *p* < 0.01. ($) for the significance of differences between LYC + DON and LYC + DON + 4-MBC, $ *p* < 0.05, $$ *p* < 0.01.

We further verified whether LYC could alleviate DON-induced activation of NLRP3 inflammasome via the ERK pathway through relevant experiments. As shown in Figure 7, DON induced an increase in NF-κB, NLRP3 inflammasome-related factors IL-1β, IL-18, Caspase-1, and ASC (*p* < 0.01) compared with the CON group, while LYC alleviated the increase in DON-induced inflammatory factors (*p* < 0.01). In addition, after 4-MBC addition, the mitigative effect of LYC was reduced compared with the LYC + DON group (*p* < 0.05, *p* < 0.01). These results suggested that in the model of DON-induced IPEC-J2 cell damage, LYC treatment can attenuate NLRP3 inflammasome activation by targeting ERK.

## 4. Discussion

**Figure 8:**
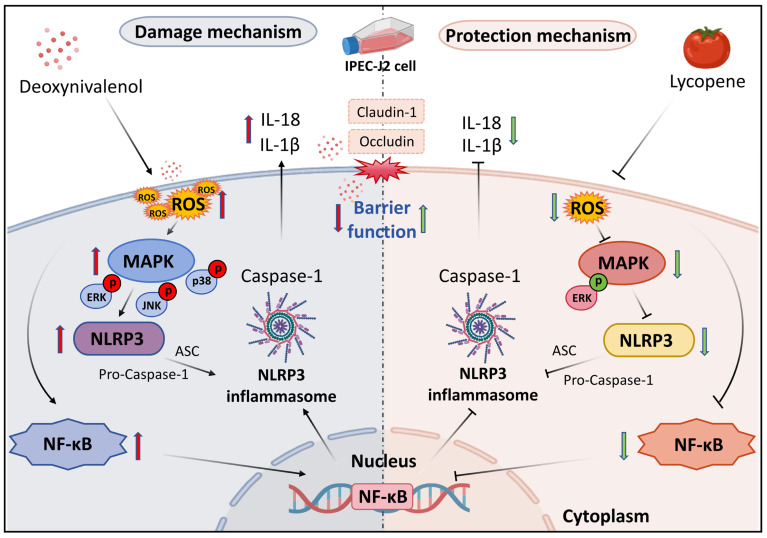
LYC inhibited DON-induced intestinal barrier dysfunction and NLRP3 inflammasome activation by targeting ERK in IPEC-J2 cells. Red arrows indicate promotion/activation, while green arrows indicate inhibition/alleviation.It is worth mentioning that the NF-κB pathway is the key factor in NLRP3 inflammasome activation and assembly [57]. NLRP3 inflammasome activation can recruit ASC (the inflammasome adaptor protein) and interact with Caspase-1. Subsequently, the secretion of IL-18 and IL-1β can be promoted by the activated Caspase-1 [58,59]. Studies have certified that caffeine inhibited the NLRP3 inflammasome activation by suppressing the NF-κB pathway [60]. A recent study has established that DON, alone or combined with Fumonisin B1, can induce enterotoxicity by promoting increases in IL-18, TNF-α, and IL-6, as well as NLRP3, Caspase-1, ASC, and IL-1β [7,61]. In addition, a low dose of DON feed for mice and piglets can aggravate ETEC-induced NLRP3 inflammasome activation, leading to intestinal inflammation and barrier dysfunction [62]. Our findings were similar to previous studies. The results demonstrated that DON can stimulate the expression of NLRP3 inflammasome-associated proteins (NLRP3, Caspase-1, ASC, and IL-1β) as well as the secretion of related pro-inflammatory cytokines (IL-18 and IL-1β). Considering this, LYC may alleviate inflammation by suppressing NLRP3 inflammasome activation because of its strong anti-inflammatory property. Therefore, we detected NLRP3 inflammasome-associated protein expression and related inflammatory cytokine levels after LYC addition. The results showed that LYC alleviated the rise in NLRP3 inflammasome-related protein expressions and cytokines (Caspase-1, ASC, IL-1β, and IL-18) boosted by DON, suggesting that LYC can mitigate NLRP3 inflammasome activation in DON-induced IPEC-J2 cells.

Currently, DON contamination in food has attracted widespread attention. Previous research reported that DON is widely exposed to all feedstuffs, especially in wheat or wheat by-products, and the contents were higher than in other raw materials [38]. DON exposure, even at low doses of ingestion, can lead to intestinal inflammatory injury in mice [39]. More studies confirmed that DON has a great influence on intestinal inflammation and barrier integrity [40]. LYC originates from watermelon, tomato, papaya, red guava, and other red fruits, and has strong antioxidant, anti-inflammatory, and other efficacy [41]. In this study, LYC alleviated DON-induced intestinal barrier damage and inflammation by regulating the MAPK pathway. Further experiments revealed that ERK is a potential target for LYC, alleviating DON-induced enterotoxicity (see Figure 8).

The intestinal epithelial barrier is the main barrier against DON exposure [14]. The integrity and function of tight junctions (TJs) are crucial for maintaining the intestinal barrier. Among them, Claudin-1 and Occludin are particularly essential for the function of TJs [42,43,44,45]. To date, DON-induced intestinal barrier damage has been extensively documented and investigated using the IPEC-J2 cells model [46]. Further research confirmed that the formation of TJs has a regulatory effect on intestinal barrier permeability [35,47]. Thus, in this study, we detected barrier function by using immunofluorescence staining (Claudin-1 and Occludin) and cell permeability assay. The results showed that DON exposure disrupted intestinal epithelial integrity. However, LYC treatment reversed this damage induced by DON to protect the IPEC-J2 cells’ barrier function. It is well worth mentioning that the restoration of Claudin-1/Occludin expression by LYC further supported its protective role in intestinal barrier integrity. Furthermore, oxidative stress is the main toxic event and the pivotal link in the toxic effects of DON. LYC is widely recognized for its powerful antioxidant capacity. In this study, LYC can significantly alleviate the oxidative stress in IPEC-J2 cells induced by DON, further confirming that LYC can alleviate cell damage induced by DON. Our previous study demonstrated that LYC mitigated DON-induced ROS overproduction and lipid peroxidation (MDA elevation)—key upstream triggers of ERK pathway activation, which in turn mediates TJs protein oxidative damage and intestinal barrier disruption in IPEC-J2 cells [36,48]. This antioxidant and barrier-protective effect of LYC aligns with the findings of Zhang et al. [49], who confirmed LYC’s capacity to scavenge ROS and preserve epithelial integrity in enterocytes.

NF-κB transcription factors are of great importance in regulating inflammation and immune response [50]. The secretion of cytokines such as IL-1β, IL-6, and TNF-α is mediated by the activity of the NF-κB pathway [51]. There is research that has reported that DON can activate the NF-κB pathway in IPEC-J2 cells, thereby increasing the release of IL-1β and IL-6 [52]. This is similar to our study, DON exposure promoted the inflammatory response (up-regulating TNF-α, IL-6, IL-1β, and IL-18, down-regulating IL-10) by activating the NF-κB pathway. LYC, as a common carotenoid with strong anti-inflammation, has been confirmed to have an anti-inflammation effect due to suppression of the NF-κB pathway in preventing cardiovascular diseases [53]. A recent study has found that LYC can prevent carbon tetrachloride-induced hepatic fibrosis by inhibiting autophagy caused by inflammation [54]. Furthermore, one research found that LYC can inhibit LPS-induced the rise of IL-6 secretion and NF-κB translocation but has no effect on TNF-α [55]. Another study confirmed that LYC can alleviate neuroinflammation by regulating inflammatory mediators (up-regulating IL-10 and TGF-β1, while down-regulating TNF-α and IL-1β) [56]. Our research verified that in DON-induced IPEC-J2 cells, LYC intervention suppressed the release of TNF-α and IL-6 as well as NF-κB translocation. This revealed that DON-induced NF-κB pathway activation can be inhibited by LYC.

MAPK is a crucial pathway involved in the inflammatory response [63]. A previous study found that p38 and ERK pathways are major pathways that induced IPEC-J2 cells inflammation under DON exposure [64], which is consistent with the broader body of research demonstrating that DON exposure exacerbates TJs impairment and inflammatory responses via activation of the MAPK pathway in this cell model [65]. However, in this study, the ERK pathway was focused on possibly due to differences in the treatment concentration and duration of DON, but ultimately, DON promoted the phosphorylation of the MAPK pathway and further induced the activation of inflammatory factors. This is similar to our study, in which DON induced the activation of the MAPK pathway. Notably, the latest research has found that the PGC-1α/SIRT3 pathway mediates DON-induced mitochondrial autophagy and liver injury in mice, with ROS overproduction (a key upstream trigger of MAPK activation) involved in this pathogenic process [66]. This suggested that ROS may serve as a key upstream mediator that selectively activates ERK (but not p38/JNK) to amplify the inflammatory response and exacerbate barrier dysfunction, rather than LYC directly binding to MAPK proteins to exert regulatory effects. Moreover, oxidative stress-mediated signal pathway dysregulation (including MAPK activation) has been identified as a conserved mechanism in mycotoxin toxicity [67]. Interestingly, we also found a slight difference from previous studies: DON did not induce the activation of p38 in our results, though the phosphorylation of p38 and its ratio were increased. This phenomenon differs from what Zhang et al. [64] reported, which stated that DON mainly activates the ERK/p38 pathway in IPEC-J2 cells. It is speculated that this might be related to the duration of cell treatment or the concentration of DON. But as a whole, it can be seen that DON exposure activated the MAPK pathway. Meanwhile, it is well worth mentioning that LYC blocked DON-induced phosphorylation of ERK and JNK but not p38. In general, these findings suggested that LYC may have therapeutic effects on IPEC-J2 cells via ERK or JNK pathways. We speculated that LYC alleviates DON-induced intestinal damage by indirectly inhibiting the ERK pathway via scavenging DON-induced excessive ROS (indicated by the ROS-ERK-NF-κB/NLRP3 axis, as illustrated in Figure 8), and this regulatory effect is closely linked to its inherent antioxidant capacity rather than direct interaction with MAPK molecules.

The above results concluded that in IPEC-J2 cells, LYC exhibited protective effects against DON-induced intestinal barrier dysfunction through modulating MAPK/NF-κB pathways and NLRP3 inflammasome. At the same time, we have found that LYC alleviated oxidative stress in DON-induced IPEC-J2 cells, and oxidative stress serves as a key target between MAPK activation and inflammatory response. This “ROS–ERK–inflammation” regulatory axis (analogous to the axis identified in aflatoxin B1-induced injury, where aflatoxin oxidase CotA alleviates damage by disrupting this axis [68]) has conserved biological significance across species, supporting the translational potential of LYC’s protective mechanism from this in vitro cell model to in vivo livestock and even human gut injury scenarios. Although the accumulation of ROS is not the main focus of this report, this finding provided a potential upstream mechanism for the regulation of the MAPK pathway by LYC as observed in our subsequent experiments. To further study the key pathway of LYC antagonizing DON toxicity in IPEC-J2 cells, the MAPK pathway was selected for in-depth research. We examined how these inhibitors (SB203580, SCH772984, or SP600125) affect cell viability together with DON. The results pointed out that when DON and MAPK inhibitors were treated separately, inhibiting ERK can significantly increase cell viability, but there was no difference between SB203580 and SP600125. The concentrations of MAPK pathway inhibitors were determined by previous studies [69]. Compared to the results previously shown in Figure 4, these new findings further confirm that ERK is the primary pathway through which IPEC-J2 cells are damaged under DON exposure. The results in Figure 4 illustrated the impact of DON on the MAPK signaling pathway. In summary, the aforementioned results indicated that DON primarily initiated the MAPK signaling pathway by activating the key ERK pathway. In order to more accurately understand the pathway of LYC in protecting IPEC-J2 cells from inflammation and barrier injury induced by DON, we next chose ERK activators (4-MBC) to further investigate the protective mechanism of LYC. We first selected a suitable concentration of 4-MBC. Our results showed that 0–0.3 μM 4-MBC did not affect cell viability, while the cell viability was lower when the concentration was between 0.4 μM and 10 μM. Therefore, we selected a 4-MBC concentration of 0.3 μM for subsequent experiments. We further detected the IPEC-J2 cells barrier by intestinal permeability assay and immunofluorescence staining. In addition, the NF-κB pathway and NLRP3 inflammasome-related proteins and genes were also detected. The results are that barrier function damage and inflammation induced by DON can be reversed by LYC and aggravated by 4-MBC. In summary, LYC could inhibit DON-induced NF-κB/NLRP3 inflammasome activation via regulating the ERK pathway in IPEC-J2 cells.

## 5. Conclusions

In summary, DON caused inflammation and barrier damage by activating MAPK pathway, NF-κB translocation, and NLRP3 inflammasome in IPEC-J2 cells. LYC intervention can alleviate the DON-induced enterotoxicity, inhibiting MAPK/NF-κB pathways and NLRP3 inflammasome activation. This research further confirmed that LYC supplement targeting of ERK can be exploited to prevent DON-induced intestinal inflammation and thus treat DON-induced intestinal diseases. While our data were generated in the IPEC-J2 in vitro model, the identified mechanism has potential for in vivo translation: the ERK/NF-κB/NLRP3 axis mediating DON-induced gut injury is evolutionarily conserved in mammals, and LYC’s inherent antioxidant and anti-inflammatory properties can be exerted via dietary supplementation, supporting its utility for mitigating DON-induced enterotoxicity in livestock and other susceptible organisms. Therefore, our findings provide an idea that LYC could serve as a novel therapeutic agent to mitigate DON-induced intestinal damage, with the effective in vitro LYC: DON ratio (30 μg/mL:0.5 μM) observed in IPEC-J2 cells, while the optimal ratio for in vivo therapeutic/prophylactic use requires further investigation.

## Data Availability

The original contributions presented in this study are included in the article/Supplementary Materials. Further inquiries can be directed to the corresponding authors.

## Supplementary Materials

The following supporting information can be downloaded at: https://www.mdpi.com/article/10.3390/antiox14121513/s1, Table S1: Primer sequence for each target gene.
