# Supplementary material for: Lycopene Protects Deoxynivalenol-Induced Intestinal Barrier Dysfunction and NLRP3 Inflammasome Activation by Targeting the ERK Pathway
Source: Antioxidants (Basel). 2025 Dec 17;14(12):1513. doi: 10.3390/antiox14121513 (PMC12729457; doi:10.3390/antiox14121513)
Supplement: Supplementary file 1 [file antioxidants-14-01513-s001.zip › antioxidants-4020515-supplementary.pdf]

Table S1 Primer sequence for each target gene

Target gene	Forward primer (5’-3’)	Reverse primer (5’-3’)	Gene accession number
β-actin	CTGCGGCATCCACGAAACTC	AGGGCCGTGATCTCCTTCTG	NM_001444420.1
IL-6	GGCTGCTTCTGGTGATGG	AGAGATTTTGCCGAGGATGTA	NM_214399.1
IL-10	AGTGCCTTTAGCAAGCTCCAA	GAGTCGTCATCCTGGAAGGTT	NM_214041.1
TNF-α	CGTTGTAGCCAATGTCAAAGCC	CTCTGGCAAGGGCTCTTGATG	NM_214022.1
NLRP3	TACTTGGGTGAAAATGCCCT	CAGAATTCACCAACCCCAGT	NM_001256770.2
CASP1	CAGGAGTCCTCGAACTCTCCACAG	GGCTCTGAAGACGCAGGCTTAAC	NM_214162.1
ASC	AGATGAAGCTGCTCTCAGTG	GAAGTGCAGTGCTGGTTTG	XM_003124468.5
IL-18	GCTGAAAACGATGAAGACCTG	ATGGTTACTGCCAGACCTCTA	NM_213997.1
IL-1β	GCCCTGTACCCCAACTGGTA	CCTCTGGGTATGGCTT	NM_001302388.2
NF-κB	AGTACCCTGAGGCTATAACTCG	TGAGAAGTCCATGTCCGCAAT	NM_001114281.1
